# Photoinduced hydrogen release from hydrogen boride sheets

**DOI:** 10.1038/s41467-019-12903-1

**Published:** 2019-10-25

**Authors:** Reiya Kawamura, Nguyen Thanh Cuong, Takeshi Fujita, Ryota Ishibiki, Toru Hirabayashi, Akira Yamaguchi, Iwao Matsuda, Susumu Okada, Takahiro Kondo, Masahiro Miyauchi

**Affiliations:** 1 Department of Materials Science and Engineering, Tokyo Institute of Technology, Tokyo, 152-8552 Japan; 2 Department of Physics, Faculty of Pure and Applied Sciences, University of Tsukuba, Tsukuba, Ibaraki 305-8571 Japan; 3 School of Environmental Science and Engineering, Kochi University of Technology, Kochi, 782-8502 Japan; 4 Graduate School of Pure and Applied Sciences, University of Tsukuba, Tsukuba, 305-8573 Japan; 5 Institute for Solid State Physics, University of Tokyo, Kashiwa, Chiba 277-8581 Japan; 6 Department of Materials Science and Tsukuba Research Center for Energy Materials Science, Faculty of Pure and Applied Sciences, University of Tsukuba, Tsukuba, 305-8573 Japan; 7 Materials Research Center for Element Strategy, Tokyo Institute of Technology, Yokohama, 226-8503 Japan

**Keywords:** Photochemistry, Materials for energy and catalysis, Two-dimensional materials

## Abstract

Hydrogen boride nanosheets (HB sheets) are facilely synthesized via ion-exchange treatment on magnesium diboride (MgB_2_) in an acetonitrile solution. Optical absorption and fluorescence spectra of HB sheets indicate that their bandgap energy is 2.8 eV. According to first-principles calculations, optical absorption seen at 2.8 eV is assigned to the electron transition between the σ-bonding states of B and H orbitals. In addition, density functional theory (DFT) calculations suggest the other allowed transition from the σ-bonding state of B and H orbitals to the antibonding state with the gap of 3.8 eV. Significant gaseous H_2_ release is found to occur only under photoirradiation, which causes the electron transition from the σ-bonding state to the antibonding state even under mild ambient conditions. The amount of H_2_ released from the irradiated HB sheets is estimated to be 8 wt%, indicating that the sheets have a high H_2_-storage capacity compared with previously reported metal H_2_-storage materials.

## Abstract (web summary)

The development of lightweight and safe hydrogen carrier materials is desirable for hydrogen energy storage and release. Here, authors prepare hydrogen boride nanosheets and observe hydrogen gas production induced by light irradiation.

## Introduction

New classes of two-dimensional (2D) materials are the subject of considerable research and development efforts for functional applications, including electronics, energy, environment, bioscience, and catalysis, similar to graphene^1–3^ and chalcogenide nanosheets^4,5^. Among the various candidates as a new class of 2D nanosheets beyond graphene^6^, borophene, which is composed of B atoms arranged in a single monoatomic layer, was theoretically predicted to have unique mechanical and electronic properties^7^ and was successfully synthesized on Ag(111) under ultrahigh-vacuum conditions^8^. Additionally, borophane, i.e. hydrogenated borophene, which is terminated with hydrogen on a hexagonal boron nanosheet, was theoretically predicted to have sufficient H_2_ storage, unique mechanical and electronic properties^9–12^. To extend the studies on these B-based 2D nanosheets, the development of a simple and facile fabrication process, e.g., Scotch-tape exfoliation of graphene, is essential^1^. Recently, Jasuja et al.^13^ reported the synthesis of boron-based nanosheets via exfoliation from magnesium diboride (MgB_2_) under ultrasonication in water^14^. However, its chemical composition was quite complicated, because it would be partially oxidized and still contained magnesium ions on its surface^13^. To understand the basic electrical and/or optical properties of this new class of nanosheets, the synthesis of well-defined and highly pure materials is indispensable for the development of various applications. Recently, our group established the synthesis of the hydrogen boride nanosheets (HB sheets) with a well-defined chemical composition using a facile ion-exchange technique in an organic solvent through an exfoliation process^15^. Interestingly, our HB sheets produced gaseous H_2_ under heat treatment at 150–1200 °C and exhibited a semiconductive photoabsorption property^15^. According to previous theoretical and experimental studies, the HB sheet is a promising material for H_2_ storage because its gravimetric H_2_ storage capacity vs. the light boron element is quite high (8.5 wt%)^9,15^. The HB sheet has more advantages than conventional H_2_-storage systems such as metal-based materials and high-pressure containers owing to its high H_2_ capacity and safe operation.

Herein, we report efficient H_2_ release from HB sheets using photon energy under extra mild conditions, i.e. the ambient room temperature and atmospheric pressure. We calculated the electronic structure of the HB sheets using density functional theory (DFT) and investigated their optical properties to examine the relationship between the electronic structure and the H_2_-release property.

## Results

### Characterization

The HB sheets were synthesized via a wet chemical exfoliation method. The starting material was magnesium diboride (MgB_2_), in which Mg ions were intercalated between each B layer, forming a honeycomb structure with *sp*^2^ bonds. MgB_2_ powder and cation-exchange resin were introduced into an acetonitrile solution, followed by stirring, where Mg^2+^ ions of MgB_2_ were exchanged with protons from the resin. This procedure was conducted at room temperature and the atmospheric pressure through N_2_ bubbling. After 3 days of stirring, the remaining MgB_2_ and resin were removed via filtration. Finally, we could obtain yellow powder of HB sheets after the filtrate was dried under reduced pressure at 313 K.

**Fig. 1:**
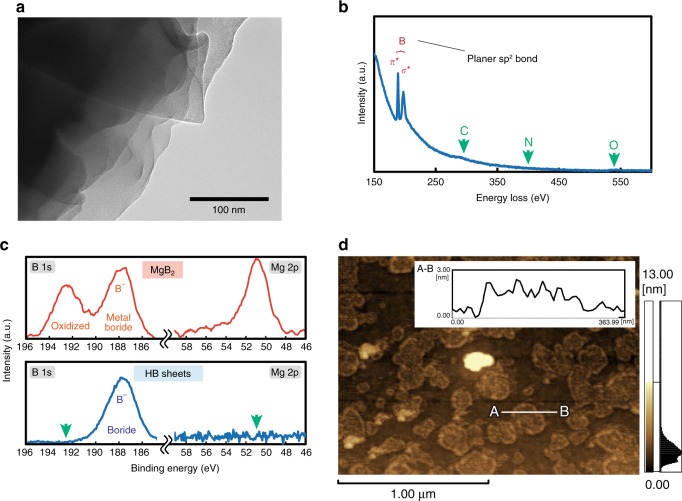
Characterization of HB sheets. **a** TEM image and **b** electron energy loss spectroscopy (EELS) of HB sheets, **c** XPS results for MgB_2_ and HB sheets, and **d** AFM image of HB sheets on mica substrate and line profile between points A and B in AFM image

A transmission electron microscopy (TEM) image of the HB sheets is shown in Fig. 1a. Thin and wrinkled structures were observed. Electron energy-loss spectroscopy (EELS) revealed only double peaks of B, indicating that the B atoms of the HB sheets retained their planar *sp*^2^ bonds even after the cation exchange (Fig. 1b)^16,17^. X-ray photoelectron spectroscopy (XPS) revealed that the B maintained its negative-charge state and that the Mg signal disappeared after exfoliation (Fig. 1c), suggesting that cations are replaced with protons. Fourier-transform infrared (FT-IR) spectroscopy revealed the formation of B–H and B–H–B bands (see Supplementary Fig. 1). The chemical composition of HB sheets was previously determined by the thermal desorption mass spectroscopy^15^. Specifically, based on the weight of the sample and quantitatively estimated desorbed hydrogen amount (using calibration made by standard sample measurements), we have estimated the ratio as H:B = 1:1. In this estimation, we corrected the effect of a small amount of impurity (boric acid) using its decomposed product amount of desorbed water at ∼400 K (mass number 18) with control experiment. Importantly, the H/B ratio obtained from HB samples with different lot with different amount of impurity always shows the same value of H:B = 1:1 after the correction^15^. Additionally, we obtained an atomic force microscopy (AFM) image of HB sheets coated on an atomically flat mica substrate. The thickness of the HB sheets was less than a few nanometers, as shown in Fig. 1d, indicating that the HB sheets had a multi-layered nature rather than a single layer.

**Fig. 2:**
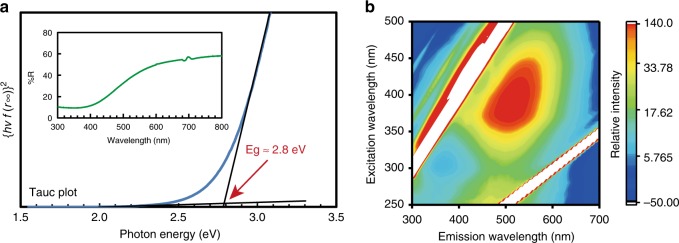
Optical properties of HB sheets. **a** Absorption and **b** 3D fluorescence spectra of HB sheets

Next, we investigated the optical absorption and fluorescence emission of the HB sheets and the results are shown in Fig. 2. According to the optical absorption spectrum (Fig. 2a), the bandgap energy of HB sheets was estimated to be 2.8 eV. The three-dimensional (3D) fluorescence spectrum is shown in Fig. 2b, and the fluorescence signal excited by 2.8 eV (~440 nm) was consistent with the absorption spectrum. Noteworthy, the fluorescence emission signal was terminated when the excitation wavelength was shorter than 350 nm. These results indicate that the unoccupied band dispersion of HB sheets is 0.6 eV, which is relatively narrow as compared to conventional semiconductors.

### DFT calculation

**Fig. 3:**
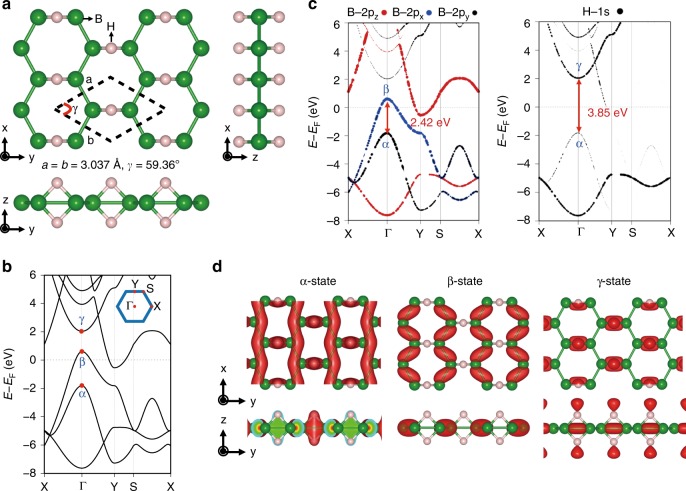
First-principles calculations for an HB sheet. **a** Top and side views of the optimized atomic structure of the HB sheet. The bonds drawings are defined by the interatomic distance, and the unit cell is indicated by the dashed lines. **b** Electronic band structure of the HB sheet and the Brillouin zone. **c** Projected band structures of B- and H-originated orbitals. **d** Square-wave function distribution of the α, β, and γ states at the Γ-point in panel **b**

To understand the optical property of the HB sheets, we performed first-principles DFT calculations on the model of the HB sheet (Fig. 3a) based on our previous characterization using an X-ray pair distribution function analysis together with Fourier-transform infrared reflection spectroscopy, XPS, and ultraviolet absorption spectroscopy^15^. The calculated model in the present study is consistent with the previous works^15,18^. The calculation was performed based on the DFT framework^19,20^ using the Quantum Espresso code^21,22^ with the semi-local Perdew, Burke, and Ernzerhof (PBE) exchange-correlation functional^23^. The norm-conserving SG15 pseudopotentials^24^ were used to describe the electron–ion interaction for B and H elements and the Brillouin-zone integration was sampled by the Monkhorst-Pack (MP) scheme^25^. The wave functions were expanded using plane-wave basis sets with cutoff energies of 100 Ry (1360 eV) in which the converged geometry and energy-band results were obtained. According to the results for the band structure and state-to-state transition probabilities (Fig. 3b–d, Table 1), the HB sheet has two major transitions, i.e. the α → β transition (2.42 eV) and the α → γ transition (3.85 eV), as shown in Fig. 3c. The former transition is attributed to the experimentally observed optical absorption. Interestingly, the HB sheet involves optical allowed transition induced by 3.85 eV photon energy (∼320 nm). The α state, which is shown in Fig. 3, is the σ-bond that consists of B 2*p*_*y*_ and H 1*s* atomic orbitals, and the γ state is the σ-antibonding of the α-state. In contrast, the origin of the β state is the σ-bond of B 2*p*_*x*_ atomic orbitals. The expected probabilities of each transition calculated via DFT are shown in Table 1. Semi-local functionals such as PBE often underestimate the bandgap^26^. Thus, we performed energy-band calculations of the HB monolayer with different versions of hybrid Heyd–Scuseria–Ernzerhof (HSE) and B3LYP exchange-correlation functionals, as shown in the Supplementary Information (Table 1). The transition energies (energy gap) between α-, β-, and γ-states at the Γ-point of the HB monolayer increased significantly from 2.4 and 3.8 eV of the PBE functionals to 2.69 (2.54) and 4.5 (4.93) eV of the HSE03 (B3LYP) functionals, respectively. However, it was reported that the hybrid exchange-correlation potentials such as HSE and B3LYP do not accurately describe electronic properties such as MgB_2_^18^. Moreover, the present calculated result for the HB sheet using PBE functionals is consistent with that of a recent report^27^. Furthermore, we calculated the bilayer structures of the HB sheet since the AFM image indicated that the HB sheets existed had a multi-layered rather than a mono-layered nature. Supplementary Fig. 2 shows top and side views of optimized atomic structures, the energy band of the HB monolayer, and four possible stacking HB bilayers. Consequently, the HB layer is weakly bound together via the van der Waals interaction, indicating that the electronic property of HB sheets exhibits like a mono-layered characteristic. These calculation results indicate the HB sheets have unique two band structure.

**Table 1:** Energy gaps and probabilities of state-to-state transition in three *x*, *y*, *z* directions from the α-state to β-state and α-state to γ-state at the Γ-point

Transition	Δ*E*	*P*_*x*_	*P*_*y*_	*P*_*z*_
α → β	2.42 eV	0.499	1.60 × 10^–5^	1.11 × 10^–5^
α → γ	3.85 eV	1.45 × 10^–5^	0.464	0.036

### H_2_ release property

**Fig. 4:**
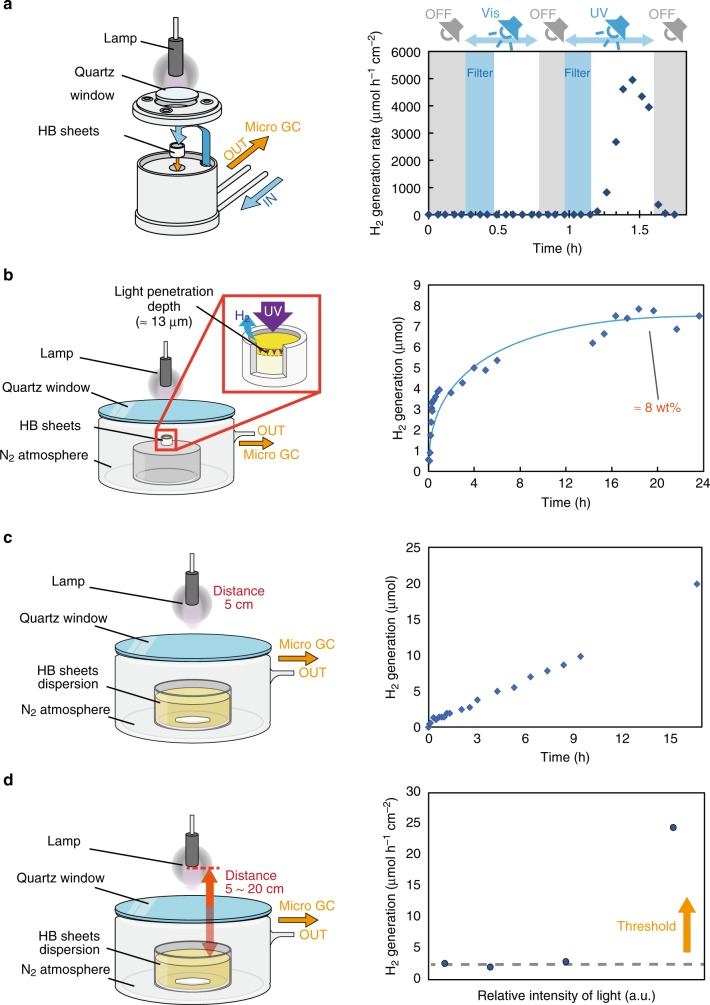
Photoinduced H_2_ release properties of HB sheets. **a** Schematic illustrations of the experimental setup and H_2_ production under different light sources in a flow reactor; **b** amount of H_2_ produced under UV irradiation, where the corresponding amount of HB sheets was estimated by the light penetration depth in HB sheets; **c** H_2_ production under UV light from HB sheets dispersed in acetonitrile; **d** light intensity dependence on H_2_ production from dispersed HB sheets, respectively. Each plot in panel **d** was measured under the distance between light source and sample at 20, 10, 6.6, 5.0 cm, respectively. Light irradiation condition in panels **b** and **c** was 5 cm distance between light source and sample

Next, we investigated the H_2_-release property of the HB sheets in a flow-type reactor through the excitation corresponding to the two bands that indicated by DFT calculations (See Supplementary Fig. 3). A mercury–xenon lamp (200 W Hg–Xe lamp as a UV light) induced the 3.8 eV transition, while a xenon lamp (150 W Xe lamp as a visible light) induced the 2.8 eV transition. Further, we used a cutoff filter (Y-490, cutoff below 490 nm) with these light sources, which led to excitation of neither transition, as a control. Figure 4a shows the results for H_2_ production under the visible light and UV light with and without the cutoff filter. Significant release of H_2_ gas from the HB sheets only occurred under the UV light (Hg–Xe lamp) without the cutoff filter, which induced the α → γ transition. On the other hand, H_2_ was not released under visible-light irradiation using the Xe lamp, although the HB sheets absorbed the visible light. These results strongly indicate that the H_2_ release is driven by the α → γ transition rather than α → β. The H_2_ release exhibited a rapid response to the on–off procedure of light irradiation, excluding the contribution of the heat effect. Our experimental and theoretical investigations suggest that H_2_ release is driven by the excited electrons in the antibonding state of the H orbital, causing the self-reduction of protons and the generation of H_2_, as shown in Supplemental information (see Supplementary Fig. 4). According to the theoretical calculation by Abtew et al.^9^ the hydrogen bonding strength with boron would be weakened by the electron doping into the antibonding state of H orbital in hydrogenated borophene, which is consistent with our experimental and theoretical results in terms of the relationship between the electron filling at the antibonding states and the bond strength. The excited electrons in the antibonding state of HB sheets are used for hydrogen production, thus the fluorescence emission by the recombination of electron–hole pairs between α and γ states is not observed in Fig. 2b. The previous studies reported multi-fluorescence emissions from boron-based nanosheets^28,29^, indicating that the photo-emission property from HB sheets would strongly depend on defects and/or surface functionalization. In addition, we checked the production of gas-phase boron species by mass spectroscopic analysis (see Supplementary Fig. 5). The formation of gas-phase boron species such as BH_3_ and B_2_H_6_ was found to be negligible or slightly seen at the beginning of light irradiation. We could temporarily detect the water and acetonitrile at the beginning of the light irradiation owing to the desorption of these species, but H_2_ was continuously evolved.

To investigate the amount of H_2_ released with respect to the unit weight of the HB sheets, we used a closed reactor, as shown in Fig. 4b. The concentration of H_2_ became saturated around 7.5 μmol, corresponding to 8 wt% of the HB sheets, by considering the optical light penetration depth in the HB sheets (∼13 μm as explained in the Experimental section). The H_2_ capacity of a unit cell of the HB sheets was 8.5 wt%, thus the amount of H_2_ released from the HB sheets was close to the theoretical storage capacity. It is noteworthy that the almost all of the terminated H ions of the HB sheets were reduced to H_2_ molecules under photon irradiation under extra mild conditions. The HB sheets consist of a few nanometers ultrathin structure, thus, almost all of the surface H ions are photoactive species for H_2_ production. This is a significant advantage compared with H_2_ storable bulk metal alloys, whose H_2_ capacities are less than a few mass percent versus the bulk alloy^30^. Conventional H_2_-storage materials are composed of metal, whose Fermi level is located in an occupied band; thus, photoinduced H_2_ release has never been observed in these bulk metal materials. It is noteworthy that the HB sheets exhibit a semiconductive light absorption property and a high H_2_ capacity, because H ions are terminated on the surface of the nanosheet. Therefore, we expect that HB sheets can be used for safe, lightweight, and photoresponsive H_2_ carriers.

Our HB sheets could be highly dispersed in a solvent such as alcohol or acetonitrile, thus, we investigated the H_2_-release property of HB sheets dispersed in acetonitrile (Fig. 4c). H_2_ was released at a constant rate under light irradiation even with the dispersion system. This indicates that our HB sheets can be utilized as H_2_ carriers in liquid-phase systems in addition to gas-phase systems, which enables great advantage for practical use. Using this liquid system setup, we can exclude the photothermal effect on the hydrogen release property since the boiling point of acetonitrile is 82 °C. The actual temperature of acetonitrile under UV light irradiation was 28 °C, implying that the hydrogen evolution from HB sheets was driven through the electron transition by photons rather than heat generation. Further, we investigated the light intensity dependence on the H_2_-release property for the liquid-phase dispersed system by changing the distance between the light source and the samples (Fig. 4d). The H_2_ molecules were released under certain photon-flux threshold, indicating that the density of electron–hole pairs is important for driving hydrogen evolution. This phenomenon differs from a conventional photochemical reaction under a light-limited condition in conventional photocatalysis.

Our experimental and theoretical studies in the present work strongly suggested that the excited electrons at the antibonding sites of H play an important role in the production of H_2_ from HB sheets. In addition to the reduction reaction caused by excited electrons, we discuss the photogenerated holes in the bonding state of the B orbital under UV irradiation. The generated holes in the B orbital oxidize negatively charged B to an intermediate less-charged state. We attempted to detect the intermediate state of B under photoirradiation in a sample-exchange pre-chamber equipped with an XPS apparatus through a quartz window (see Supplementary Fig. 6a). However, the HB sheets were easily oxidized by O contaminants, resulting in a positive-charge state of B (Supplementary Fig. 6b), because of the insufficient vacuum level (∼10^–4^ Pa) in the pre-chamber. However, interestingly, we detected the intermediate charged state of B (B_in_) between B^+^ and B^−^ signals in an ultrahigh-vacuum chamber (∼10^–8^ Pa) under low-energy X-ray exposure (see Supplementary Fig. 6d). We consider that the intermediate charged B states were temporarily formed under UV irradiation, leading to H_2_ generation, similar to the case of heat treatment. With UV irradiation under the ambient atmospheric conditions, amorphous or partially oxidized B would be produced after the release of H_2_. The FT-IR spectra and TEM image of HB sheets after UV light irradiation are shown in the Supplementary Information (see Supplementary Figs. 7 and 8). The B–H bonding signal (∼2500 cm^−1^) decreased significantly after light irradiation, while their sheet-like morphology was observed even after UV irradiation.

We note here that the boric acid was sometimes included in the pure HB sheets because of the incomplete separation of boric acid from HB during synthesis (e.g. Supplementary Fig. 1). To check the effect of these included boric acid on the light-induced hydrogen production, we did control experiment, i.e. we examined optical properties (absorption and fluorescence) and hydrogen release property of commercial boric acid. We confirmed that the boric acid is an ultra-widegap material and did not overlap the optical property of HB sheets. Further, no hydrogen was released from the boric acid under the same UV irradiation. Thus, we can safely exclude the effect of boric acid on the light-induced hydrogen production from HB sheets. Furthermore, the effect of water molecules on the hydrogen generation was investigated. We added HB sheets into water and evaluated their hydrogen production property (see Supplementary Fig. 9). Although the small amount of hydrogen (2.3% of HB molecules) was released through hydrolysis reaction, significant H_2_ release under UV irradiation cannot be simply explained by hydrolysis. Other data, including DFT calculations, light wavelength, and intensity dependence, support that the H_2_ molecules are released from HB sheets by photogenerated charge carriers, rather than by hydrolysis reaction or decomposition of boric acid impurities.

It is noted that the amount of released hydrogen from the irradiated HB sheets was 8 wt%. This indicates that the unique optical and physical properties presented in this study are owing to the HB sheet characteristics rather than boric acid or adsorbed water. Re-storage of hydrogen into HB sheets is one of the most significant challenges for practical application as H_2_ carriers. However, we expect the HB sheets to have the rechargeable H_2_ property, because of the capability of H_2_ to absorb amorphous B^31^. The H_2_ re-storage property of HB sheets is currently under investigation and will be reported elsewhere.

## Discussion

In the present study, significant H_2_ release was induced from HB sheets only by photon irradiation. Our HB sheets are lightweight, and their H_2_-storage capacity is higher than that of bulk alloys. The significant H_2_ release from the HB sheets was due to their 2D nanosheet structure and unique electronic band structure. This intriguing phenomenon enables us to provide a portable hydrogen generator wherever on-site using HB sheets and a light source. The results also indicate that the photogenerated charge carriers in HB sheets can initiate a chemical reaction, in addition to H_2_ production. We expect the present study to stimulate research on hydrogen boride for various fields not only for H_2_-storage applications but also for electrical and semiconductive materials, electrochemical devices, photocatalysts, etc.

## Methods

### Synthesis of HB sheets

Magnesium diboride powder (MgB_2_, 500 mg, 99%; Sigma-Aldrich, St. Louis, MO, USA) and a cation-exchange resin (30 mL, Amberlite IR120B hydrogen form; Organo Corp., Tokyo, Japan) were added to acetonitrile (99.5%, JIS special grade; Wako, Osaka, Japan) at room temperature, followed by stirring at the atmospheric pressure in an N_2_ atmosphere. After 3 days of stirring, the remaining MgB_2_ and resin were removed from the solution via a membrane filter (Omnipore 0.2 μm; Merck Millipore Ltd, Ireland). The filtrate, which was a yellow dispersion, was dried under a reduced pressure at 313 K, yielding HB sheets in the form of yellow powder.

### Characterization

Transmission electron microscopic (TEM) was performed using JEM-2100F TEM/STEM (JEOL Ltd, Japan) operated at an acceleration electron-beam voltage of 200 kV. XPS measurement was performed using JPS 9010 TR (JEOL, Ltd, Japan) with an Al Kα X-ray source (1486.6 eV). The samples were fixed on graphite tape. The absolute binding energy was calibrated according to the peaks of B 1*s* in B(OH)_3_ or the C 1*s* orbitals. Fourier-transform infrared spectroscopy (FT-IR) spectra were recorded using FT/IR-6100 (JASCO, Co., Ltd, Japan). Approximately 0.25 wt% of HB sheets was mixed with KBr powder to fabricate pellets for FT-IR measurement. UV–visible (UV–vis) diffuse reflectance spectra were recorded using a spectrophotometer (V-670, JASCO, Co., Ltd, Japan) equipped with an integration sphere unit. The bandgap of the HB sheets was calculated via extrapolation in the Tauc-plot curve of the Kubelka–Munk function. A white BaSO_4_ plate was used as a reflectance standard. The 3D fluorescence spectra of the HB sheets were recorded using a spectrofluorophotometer (F-7000; Hitachi High-Tech Science Corporation, Ltd, Japan). The measurement was conducted at room temperature in an N_2_ atmosphere. For an AFM measurement, the HB sheets were coated on an atomically flat mica substrate via spin coating using the solution of HB sheets dispersed in acetonitrile. The spin coating was conducted at 2000 r.p.m. for 20 s. AFM images were obtained in the dynamic mode using SPM-9700 (Shimadzu Corp., Kyoto, Japan) with a silicon cantilever.

### DFT calculation

First-principles total-energy calculations were performed within the framework of DFT^19,20^ by using the Quantum Espresso code^21,22^ with the Perdew, Burke, and Ernzerhof exchange-correlation functional^23^. The norm-conserving SG15 pseudopotentials^24^ were used to describe the electron–ion interaction for B and H elements. The valence wave functions were expanded using a plane-wave basis set with a cutoff energy of 100 Ry. Both the internal atomic coordinates and lattice parameters of borophane monolayer were fully optimized until the residual forces acting on each atom were less than 10^–4^ Ry/Bohr. The 1 × 1 unit cell was used for the borophane monolayer. To simulate the isolated borophane monolayer, the sheet was separated by periodic images with a vacuum region of 10 Å. The Brillouin-zone integration was sampled by the Monkhorst–Pack (MP) scheme^25^ with 42 × 42 × 1 k-point grids in the self-consistent field calculations for optimization structures and energy band structures. The state-to-state transition probabilities were estimated according to the transition dipole moment from state *i* to state *j* and were normalized by a factor of 10^5^. The exciton effect is significant in low-dimensional systems because electrostatic screening is limited. The relatively good agreement of the experimental fluorescence energies and simulation energy gaps is thus considered to be due to the error cancelation between the underestimation of the fundamental bandgap and the absence of the exciton effect in our DFT calculations. All calculations were performed on the SGI ICE XA/UV supercomputer at the Institute of Solid State Physics, The University of Tokyo, Japan.

### H_2_-release property of HB sheets

To evaluate the photoinduced H_2_-release property of the HB sheets, 20 mg of HB powder was mounted on a porous ceramic cup (0.48 cm in diameter), and the cup was placed in a flow reactor, as described in the main text (Fig. 4a). Argon gas was flowed through this reactor (10 mL/min) and passed through pores in the ceramic cup, and then the hydrogen evolution at an outlet was measured using a micro-gas chromatograph (micro-GC, 90 Micro GC; Agilent Technologies, Co., Ltd, USA) under light irradiation. Inner diameter of the ceramic cup was 0.48 cm, and irradiated area was 0.18 cm^2^. We used two types of light sources (Supplementary Fig. 3) and a cutoff filter (Y-49) which cannot transmit light with a wavelength shorter than 490 nm. We also measured the photoinduced H_2_ release from an HB sheet dispersion. HB sheets were dispersed in acetonitrile in a glass beaker, which was placed in a 500-mL closed glass cell filled with inert gas, as shown in Fig. 4c. Light irradiation was performed through a quartz window of the cell, and then the H_2_ concentration was measured using the micro-GC (90 Micro GC; Agilent Technologies, Co., Ltd, US). We investigated the light intensity dependence on H_2_ release by changing the distance between the light source and the dispersed samples (Fig. 4d).

### Estimation of light penetration depth for determination of released H_2_ amount vs. unit mass of HB sheets

To estimate the light penetration depth into the HB sheets, we fabricated a thin film of HB sheets on a transparent quartz substrate. The thickness and transmittance of this film were measured using a three-dimensional laser microscope (LEXT OLS4000; Olympus, Co., Ltd, Japan) and a spectrophotometer (V-670; JASCO, Co., Ltd, Japan), respectively. Based on these results, the absorption coefficient (*α*) was estimated according to the Beer–Lambert law. On the basis of the *α* value, we roughly estimated the light penetration depth as the distance where the light intensity was 1% that of the incident light at 360 nm because the photon density was the highest around 360 nm for the Hg–Xe lamp used in the present study. Under this condition, the light penetration depth was 13 μm. The amount of irradiated HB sheets was calculated according to the light penetration depth and the packing density of the HB sheets in a ceramic cup. The results indicated that 8 wt% of H_2_ was released from the irradiated HB sheets.

## Supplementary information

Supplementary Information

Supplementary Figure 1

Supplementary Figure 2

Supplementary Figure 3

Supplementary Figure 4

Supplementary Figure 5

Supplementary Figure 6

Supplementary Figure 7

Supplementary Figure 8

Supplementary Figure 9

Source data

## Data Availability

The data that support the findings of this study are available from the article and Supplementary Information files, or from the corresponding authors upon reasonable request.
