# Supplementary material for: Photoinduced hydrogen release from hydrogen boride sheets
Source: Nat Commun. 2019 Oct 25;10:4880. doi: 10.1038/s41467-019-12903-1 (PMC6814783; doi:10.1038/s41467-019-12903-1)
Supplement: Supplementary file 1 — Supplementary Information [file 41467_2019_12903_MOESM1_ESM.pdf]

Supplementary Information

Photoinduced hydrogen release from hydrogen boride sheets

Reiya Kawamura [1,8], Nguyen Thanh Cuong [2,8], Takeshi Fujita [3], Ryota Ishibiki [4], Toru Hirabayashi [1], Akira Yamaguchi [1], Iwao Matsuda [5], Susumu Okada [2], Takahiro Kondo [6,7*] and Masahiro Miyauchi [1*]

[1] Department of Materials Science and Engineering, Tokyo Institute of Technology.
[2] Department of Physics, Faculty of Pure and Applied Sciences, University of Tsukuba.
[3] School of Environmental Science and Engineering, Kochi University of Technology.
[4] Graduate School of Pure and Applied Sciences, University of Tsukuba.
[5] Institute for Solid State Physics, University of Tokyo.
[6] Department of Materials Science and Tsukuba Research Center for Energy Materials Science, Faculty of Pure and Applied Sciences, University of Tsukuba.
[7] Materials Research Center for Element Strategy, Tokyo Institute of Technology.
[8] These authors equally contributed to this work.

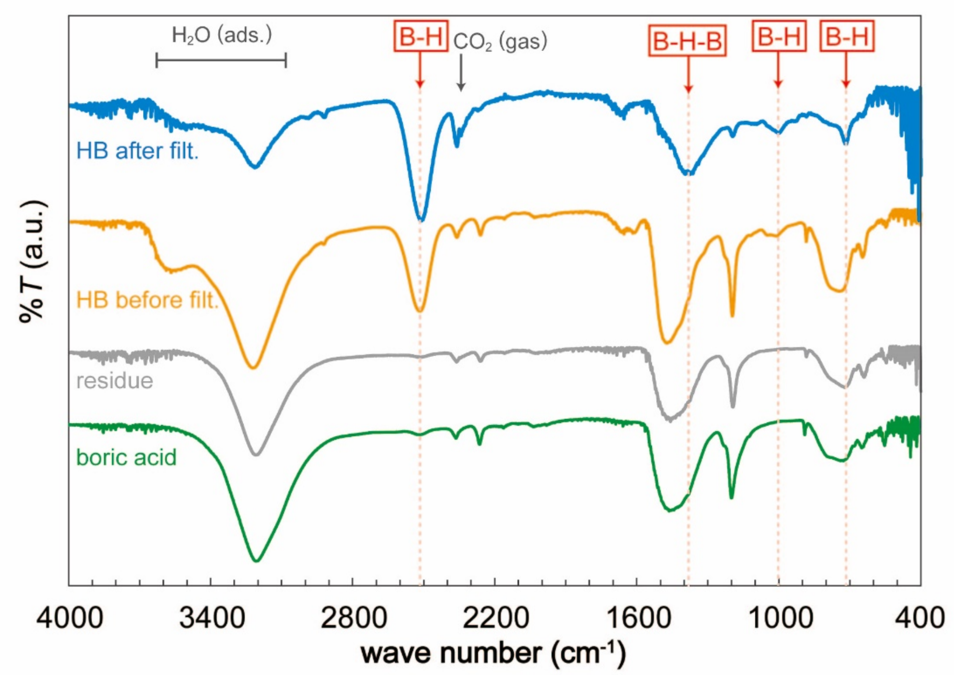

Fig. 1 FT-IR spectra of HB sheets after filtration (blue), HB sheets before filtration (orange), residue on a filter (gray), and commercial boric acid (green), respectively.

For the sample in our FT-IR measurements, boric acid residues were included after ion exchange process due to incomplete separation of boric acid from HB (see reference No. 15 in the main text). Those were thus removed by a careful filtration procedure. The broad FT-IR signal around 3200 cm^{-1} was still seen even after filtration, which is attributed to adsorbed water species, since the XPS spectrum of HB sheets clearly shows the absence of oxidized boron (positively charged boron peak at 193.2 eV) in our sample (Fig. 1(c) in the main text).

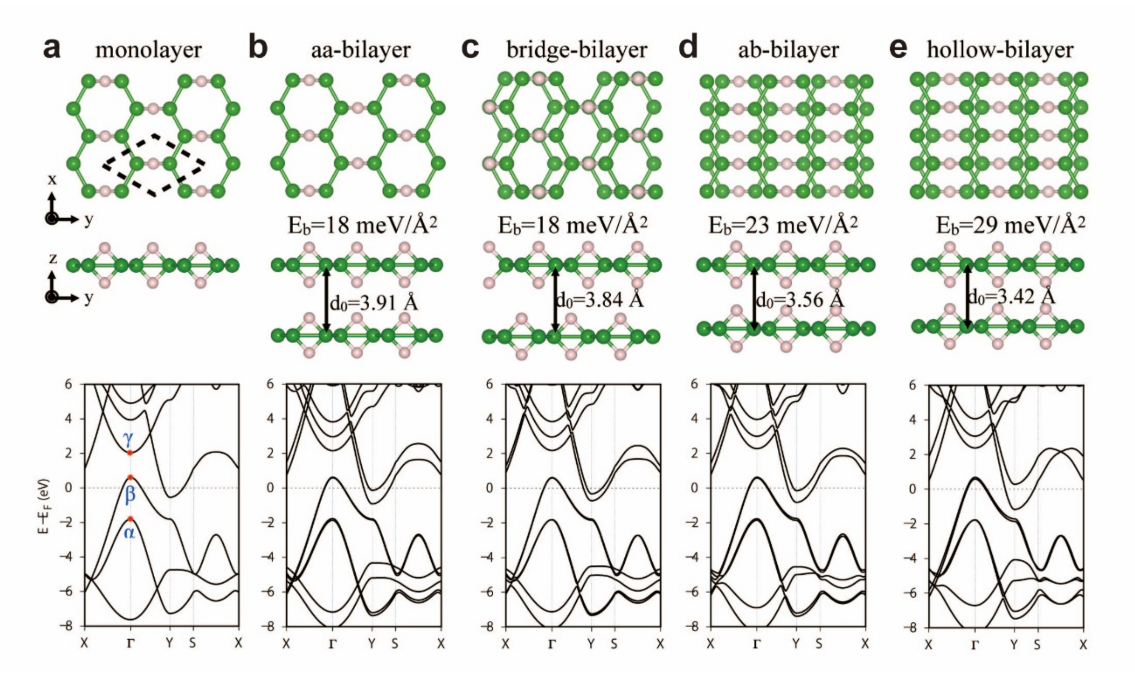

Fig. 2 Top and side views of optimized geometry and electronic energy band of (a) monolayer HB, (b) aa-stacking bilayer HB, (c) bridge-stacking bilayer HB, (d) ab-stacking bilayer HB, and (e) hollow-stacking bilayer HB. E_b is binding energy of bilayer HB with different stackings, in which $E_b = 2E_{monolayer} - E_{bilayer}$, where $E_{monolayer}$ and $E_{bilayer}$ are total energies of isolated layer and bilayer systems, respectively. d_0 is equilibrium distance between two boron layers. DFT van-der-Waals-corrected exchange-correlation functional [rev-vdW-DF2, I. Hamada, Phys. Rev. B 89, 121103(R) (2014)] is used to accurately describe the weak interaction between layers. The Fermi energy level is set to zero.

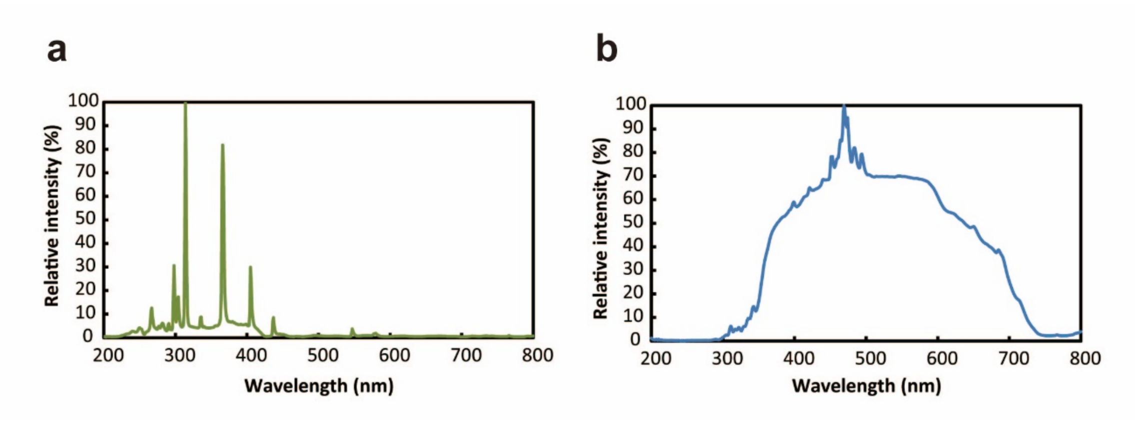

Fig. 3 Spectra of light sources. (a) Hg-Xe lamp (UV) and (b) Xe lamp (visible light).

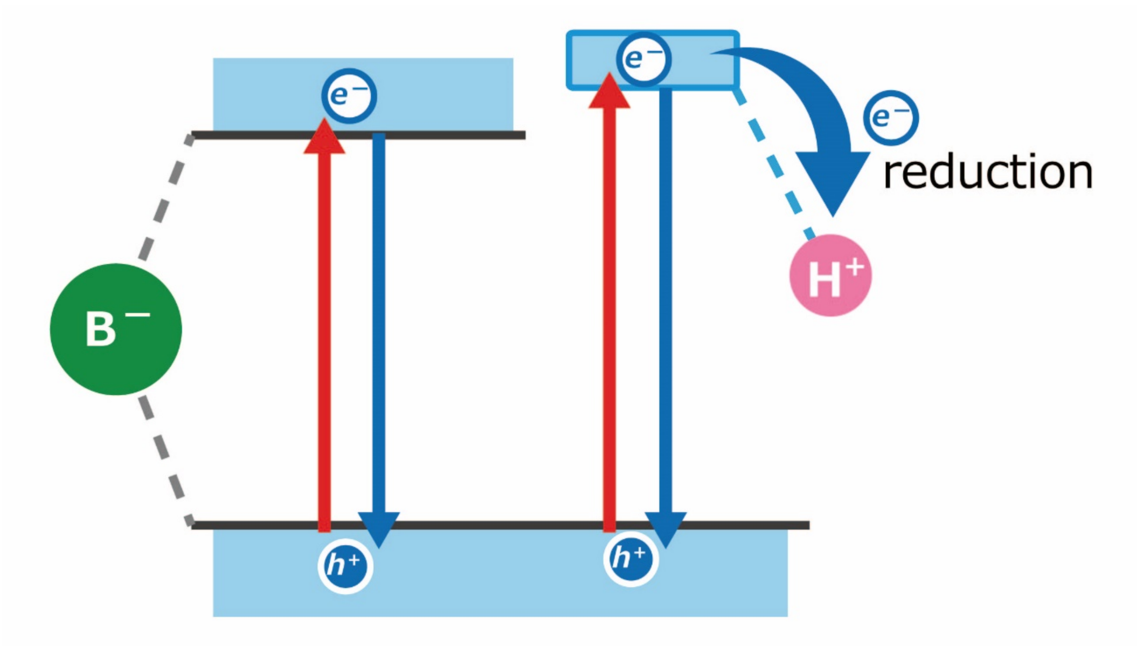

Fig. 4 Schematic diagram of the photoinduced H_2-release property of the HB sheets.

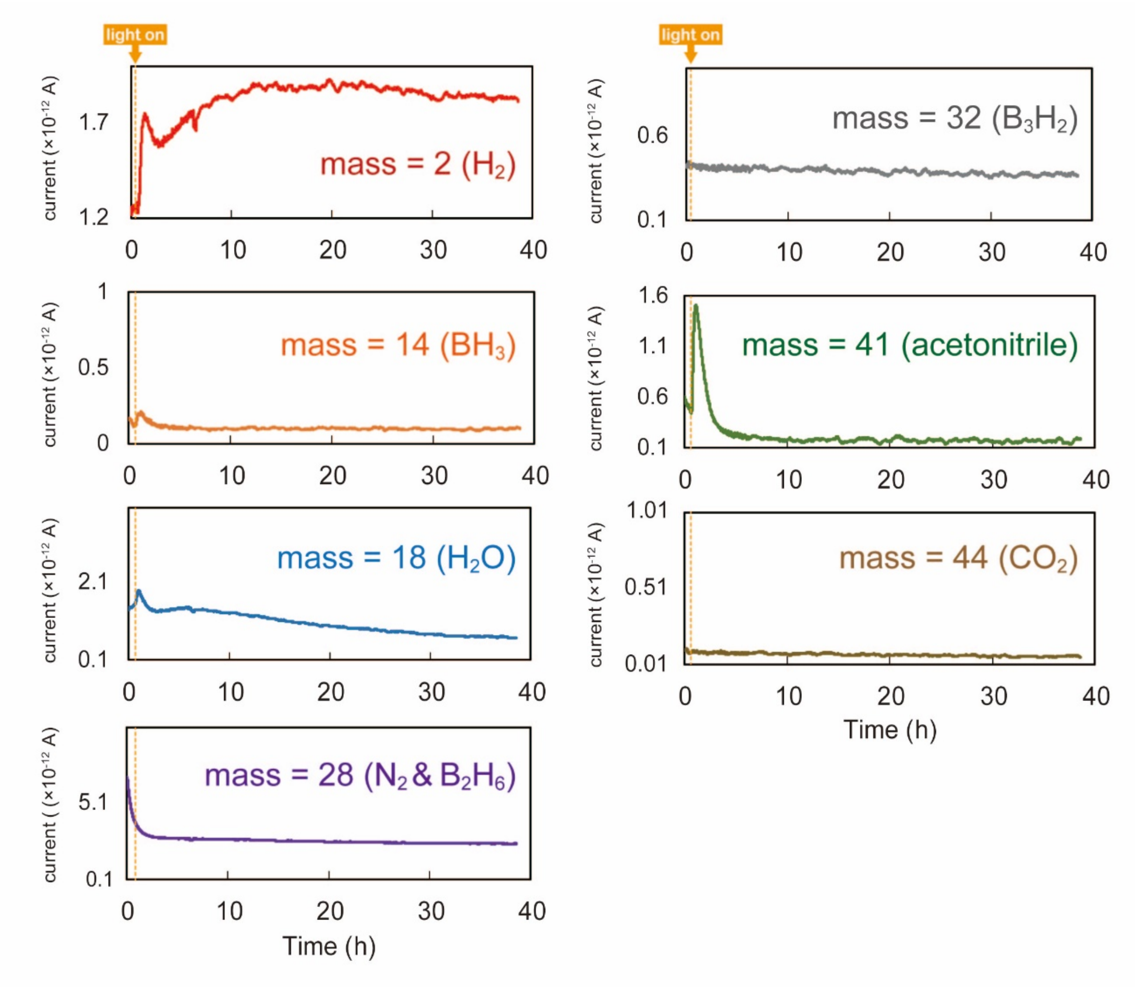

Fig. 5 Mass spectroscopic analysis of products under UV light irradiation.

Mass spectroscopy (Q-Mass) analysis was performed to relatively discuss the trend in released molecules, while the quantitative analysis of generated hydrogen was conducted using a gas chromatograph (GC) as shown in the main text. Noticeably, experimental conditions of Q-Mass and GC were different, as a flow reactor was used for Q-Mass analysis, while both flow and closed reactors were used for GC measurement.

According to the Q-Mass results in Fig. S5, hydrogen production under light irradiation was significant as compared to other gas species. The formation of gas-phase boron species such as BH_3 and B_2H_6 was found to be negligible or slightly seen at the beginning of light irradiation. We could temporarily detect the water and acetonitrile desorption. Desorption of water persists longer time as compared to acetonitrile, since the polarity and boiling temperature of water are higher than those of acetonitrile.

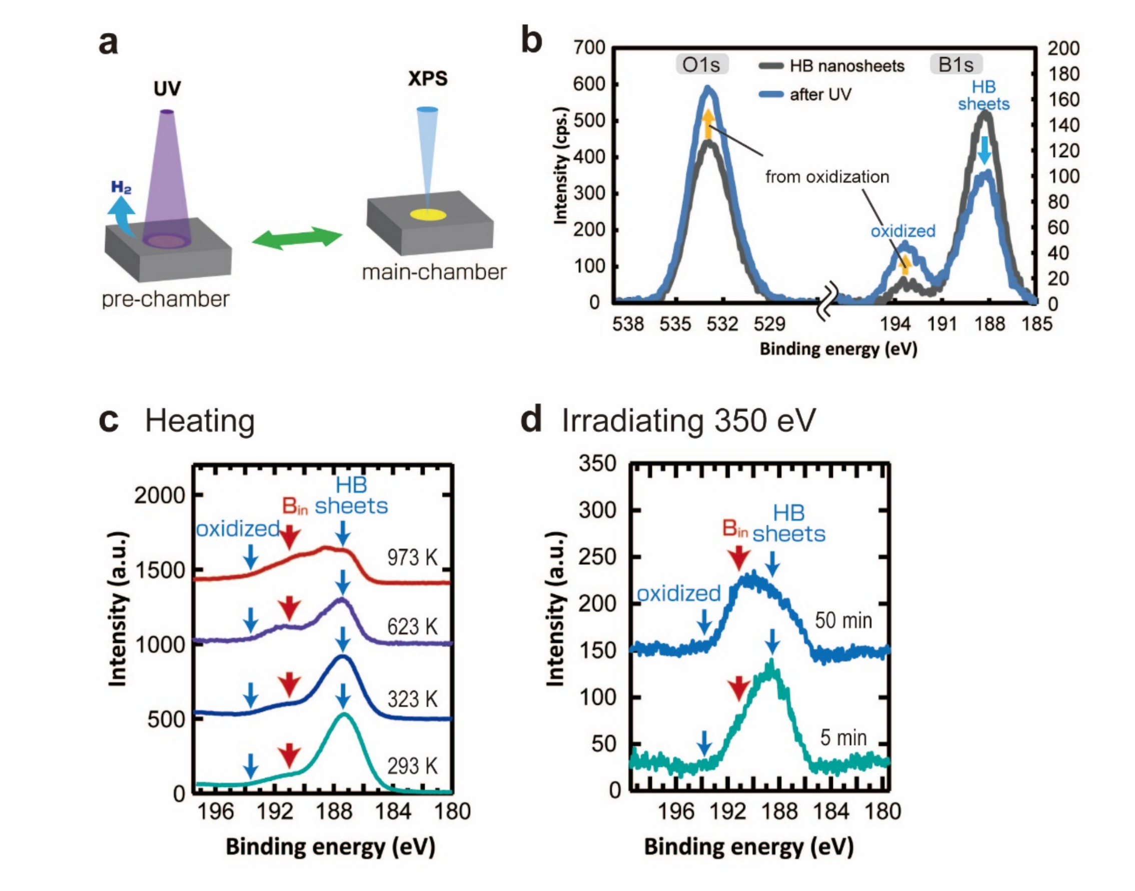

Fig. 6 X-ray photoelectron spectroscopy (XPS) analyses on HB sheets under light irradiation. (a) schematic illustration for the measurement in a sample-exchange pre-chamber equipped with an XPS apparatus; (b) XPS spectra of HB nanosheets irradiated in the pre-chamber; (c) XPS spectra of HB sheets in an ultrahigh-vacuum condition under heat treatment; (d) XPS spectra of HB sheets in an ultrahigh-vacuum condition under low-energy X-ray irradiation (350 eV).

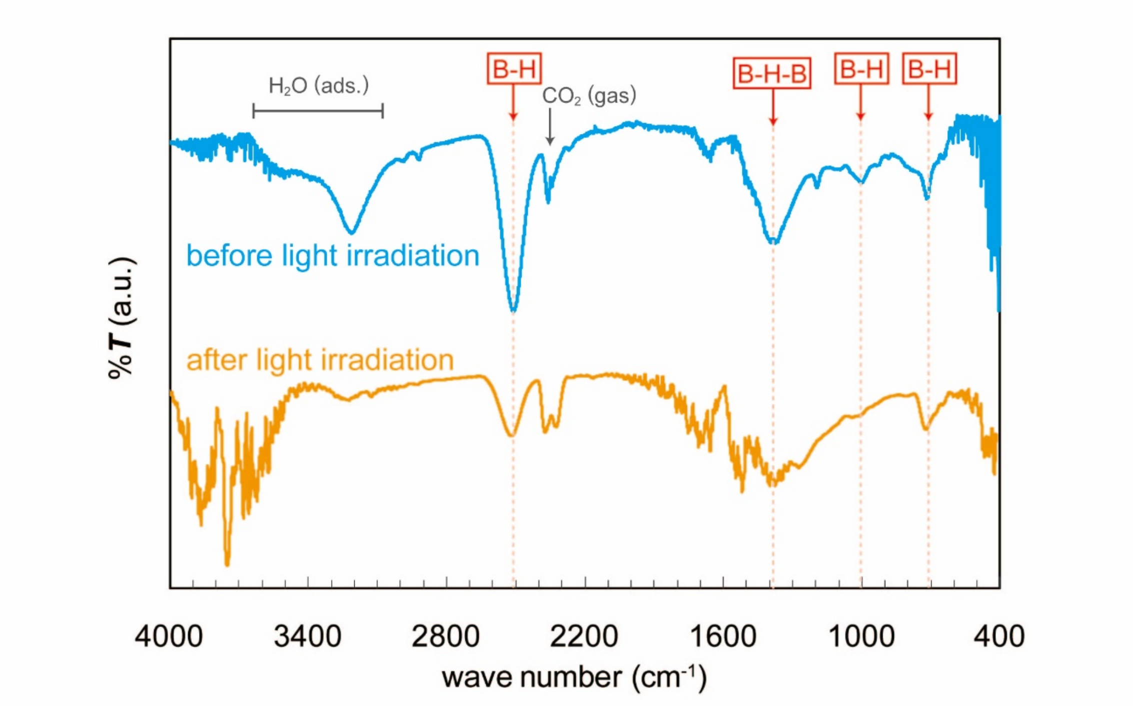

Fig. 7 FT-IR spectra for HB sheets before (blue) and after (orange) light irradiation.

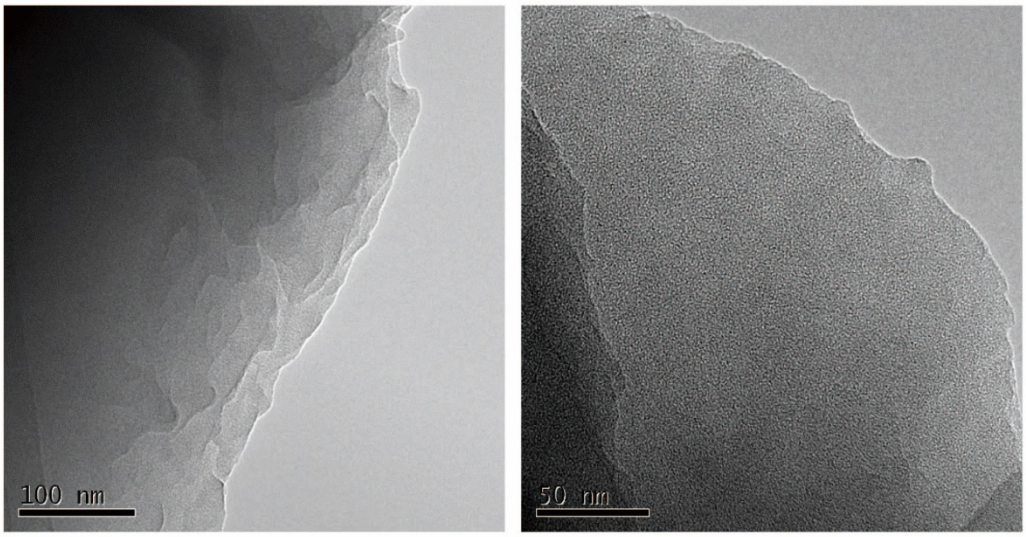

Fig. 8 TEM images of HB sheets after light irradiation for different magnification.

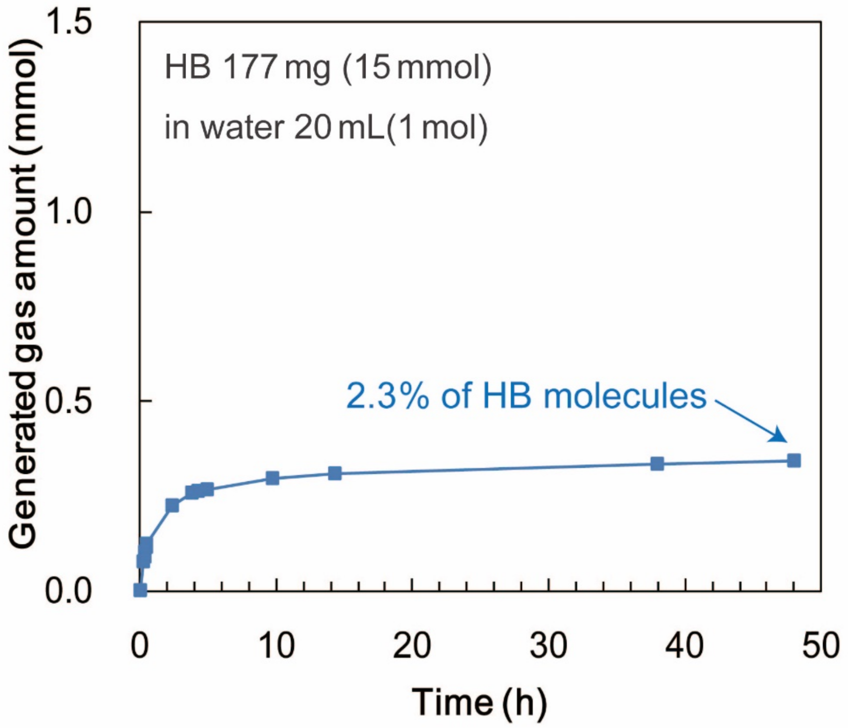

Fig. 9 Hydrogen generation from HB sheets in water. The volume of generated gas was measured by collecting it over water.

The collected gas was sampled and analyzed using a gas chromatograph, then the generated gas was identified as H_2. The result indicates that a small part of HB sheets react with water to form H_2. The detail of the limited reaction in water will be discussed in our future work. Although the hydrogen production amount under UV irradiation was micro mole order (Fig. 4 (b) in the main text), UV light penetration depth into HB sheets was quite limited (only 13 μm) because of their high absorption property, and the irradiated area on a ceramic cup was small (0.18 cm^2) as described in the main text. Based on these results, we can safely conclude that the hydrogen production amount from the irradiated HB sheets is much larger than that in water.

Table 1 Energy difference between α-, β-, and γ-states at the Γ–point of HB monolayer with semi-local (PBE) and several hybrid (HSE, B3LYP) DFT exchange-correlation functionals. It is well known that these improve the description of the energy band gap in semiconductors and insulators, but results critically depend on amount of Hartree-Fock exchange included in the HSE, B3LYP hybrid functionals.

	PBE	HSE03	HSE06	HSE12	B3LYP
$\Delta E = E_\beta - E_\alpha$	2.42 eV	2.69 eV	2.68 eV	2.69 eV	2.54
$\Delta E = E_\gamma - E_\alpha$	3.85 eV	4.48 eV	4.63 eV	4.93 eV	4.93
